# People, places, things and communities: expanding behaviour settings theory in the twenty-first century

**DOI:** 10.1098/rstb.2023.0282

**Published:** 2024-09-23

**Authors:** Marek McGann, Miranda Lucas, Cillian McHugh, Louise Barrett

**Affiliations:** ^1^ Department of Psychology, Mary Immaculate College, Limerick V94 VN26, Ireland; ^2^ Department of Psychology, University of Lethbridge, Lethbridge T1K 3M4, Canada; ^3^ Department of Psychology, University of Limerick, Limerick V94 T9PX, Ireland

**Keywords:** behavioural ecology, behaviour settings, situated cognition, embedded cognition

## Abstract

Trends and developments in recent behavioural and cognitive sciences demonstrate the need for a well-developed theoretical and empirical framework for examining the ecology of human behaviour. The increasing recognition of the role of the environment and interaction with the environment in the organization of behaviour within the cognitive sciences has not been met with an equally disciplined and systematic account of that environment (Heft 2018 *Ecol. Psychol*. **30**, 99–123 (doi:10.1080/10407413.2018.1410045); McGann 2014 *Synth. Philos.* **29**, 217–233). Several bodies of work in behavioural ecology, anthropology and ecological psychology provide some frameworks for such an account. At present, however, the most systematic and theoretically disciplined account of the human behavioural ecosystem is that of behaviour settings, as developed by the researchers of the Midwest Psychological Field Station (see Barker 1968 *Ecological psychology: concepts and methods for studying the environment of human behavior*). The articles in this theme issue provide a critical examination of these theoretical and methodological resources. The collection addresses their theoretical value in connecting with contemporary issues in cognitive science and research practice in psychology, as well as the importance of the methodological specifics of behaviour settings research. Additionally, articles diagnose limitations and identify points of potential extension of both theory and methods, particularly with regard to changes owing to the advance of technology, and the complex relationship between the individual and the collective in behaviour settings work.

This article is part of the theme issue ‘People, places, things, and communities: expanding behaviour settings theory in the twenty-first century’.

## 1. Cognitive science needs a behavioural ecology

In recent decades, cognitive science has come to a renewed appreciation of the role of the environment, of social and material context, in the processes of cognition and behaviour. The arising of ‘embedded’, ‘distributed’ and ‘situated’ approaches to cognitive science [1] has seen a push against forms of computationalism that isolate the cognitive agent from the world around it behind a screen of interpretation and information processing. Instead, we have seen the rise of avenues of research that integrate environmental dynamics into the ongoing processes of making sense of, adapting to, and working with the world [2–6].

There are a number of significant implications of this shift in perspective, some philosophical, some theoretical and some methodological.

Philosophically, the kind of embodied, embedded and enactive cognitive science that has produced these new insights appears to unsettle long- and deeply held intuitions, particularly in what can be called Western traditions [6]. The independence and autonomy of the mind from the world around it is fundamental to individualistic Western cultures, which have traditionally followed a strong separation between mind and body, mind and world (Baggs & Sanchez de Oliveira [7]). In contrast to these philosophical tenets, embodied, embedded and enactive cognitive science offers a wholesale reconceptualization of life, mind and being, with sweeping implications for scientific, medical and ethical practice [4,8–12].

Contemporary global challenges such as climate change have perhaps led to a greater recognition of our interdependence with the world around us. While some may harbour ambitions to transcend this interdependence, by leaving behind either our bodies or the Earth [13,14], the stubbornly recalcitrant science on this relationship makes it very clear that neither uploading our consciousness nor sending rockets to Mars will be successful unless we somehow bring our entire ecosystem with us [15,16]. The profundity and intimacy of our connection with our environment seem easy to dismiss but are ultimately hard to suppress: *Naturam expellas furca, tamen usque recurret* ^1^ (Horace [18]). This embedding in ecology is something that exists on the smallest scale. Immune systems, for instance, are involved less with drawing a boundary between the organism and the world than with ensuring our living bodies are well acquainted with the right microbiological crowd [19,20]. To borrow an analogy from Dewey [21], the riches and complexity of human lives remain microbiological in the way that mountains are not added on top of the ground, but are manifestations of it [22]. As organisms, we exist as manifestations of our biological ecology; as agents, we exist as manifestations of our behavioural ecology.

It will take a long time for such counter-individualist implications to work their way through to changing intuitions and common sense, and a reimagining of what it is to be a thinking being. These seismic changes may be underway, though we have seen few tremors in real terms yet.

Theoretically and methodologically, things move much quicker. Since Gibson’s seminal work [23,24], ecological psychologists have proposed that the unit of analysis in the behavioural sciences shift from the individual organism to the system of organism-and-environment [25,26]. Along with others (e.g. [27,28]; see [29] for a more comprehensive overview), they have imported into cognitive science the tools and analytical techniques of dynamical systems analysis, which have resulted in really quite dramatic transformations of how the psychological agent is conceived and investigated.

A host of theoretical resources now exist to recognize the mutuality of embodied agent and environment. These bring with them appropriate tools of observation, measurement and analysis of performance in experimental tasks or similarly circumscribed real-world performance (e.g. [25,28,30–34]). While this research acknowledges the importance of the behavioural ecosystem in the organization and production of behavioural performance, where we have not done quite so well is in the development of a comprehensive account of that behavioural ecosystem itself. Our enthusiasm for situatedness—the ecological and embedded character of psychological phenomena—must be balanced by an equally passionate enthusiasm for a scientific account of that ecology.

We suggest that there is still a good deal of work to be done on this front. However, no fewer than four bodies of extant literature are relevant here.

The first is behavioural ecology, which involves the observation of real-world behaviour contextualized within accounts of habitat and ecology. Although the models generated by behavioural ecological theorizing are broadly applicable to all animals (and indeed plants), their application to humans must contend with the complex interweaving of cultural, symbolic and social aspects of the human habitat with our evolved biological capacities. While all animals engage in niche construction to some extent, and we see the coevolution of animal and habitat, the human case brings a complexity that is challenging to discipline and systematize in a fine-grained manner. Of course, there is an entire field of study dedicated to this—our second body of literature: anthropology.

Ostensibly part of the core set of cognitive scientific disciplines [35], in reality, anthropology is the ‘missing’ discipline (e.g. [36]), having unfortunately played a limited role in the development of the science despite having much to offer [37]. Nevertheless, cognitive anthropologists have played a significant part in the development of embedded, distributed notions of cognition, perhaps most notably Hutchins [38–40] and colleagues [41]. Examining cognition as a cultural rather than an individualized, neural or personal process provides both new insight and highlights the need for a better account of that cognitive ecology. Cognitive science will not succeed without fully incorporating the insights of anthropological study. However, while the recognition of the situatedness of cognition owes much to cognitive anthropology, internal divisions within the discipline [37,42] have prevented a more systematic account of behavioural ecology from being developed. The vital importance of understanding cognitive activity within its ecosystem has been demonstrated, but how to structure a systematic account of that ecosystem remains something of an open question.

The third domain of relevant research is ecological psychology, as derived from the work of James and Eleanor Gibson [23,43,44], and those who have continued their work (e.g. [26,30,45–49]). This is invaluable research, and it is largely among researchers within this domain that we have seen the more effective development and deployment of dynamical systems analyses for behaviour in humans and animals. However, the ecology of ecological psychology is mostly considered in physical and energetic terms—the surfaces of solids, liquids and gases and arrays of ambient energy and chemicals—and how these things can provide fine guidance for the organization of behaviour. Although the Gibsons had significant interest in social and cultural aspects of psychology, there has been only a limited examination of these broader phenomena, despite the need being highlighted by several authors. Chief among them is perhaps Alan Costall, who has been highlighting the need for a fully socialized ecological psychology for a very long time [50,51]. He has certainly not been alone in doing so [47,49,52–55].

The fourth and final body of research relevant to the development of a theory of behavioural ecosystem is the subject of this theme issue.

## 2. Behaviour settings is the best available account of behavioural ecology

Behaviour settings theory represents the most powerful and finely developed account of human behavioural ecology extant in the research literature. Initially created as a means of making sense of ethnographic observational data of 16 children in a small town in rural Kansas in the early 1950s [56], it was developed, extended and empirically evaluated by the research team involved over a period of more than 25 years [57].

The team was based in Oskaloosa, KS, given the pseudonym ‘Midwest’ for the purposes of research, and so constituted the Midwest Psychological Field Station. Led by Roger Barker, the formation of the research project owed much to his colleague and wife, Louise Shedd Barker, who was initially trained as a marine ecologist. In an autobiographical essay, Roger [58, p. 507] would note that one of the principles upheld by the marine institute where Louise studied was that behavioural observations ‘explore the organism–environment relations in adjacent shores and ocean’. This ecological sensitivity was then combined with what Barker referred to as a negative ‘Aha!’ moment, while commuting across rural Illinois on a train, passing through numerous small towns and realizing that despite being a highly trained behavioural scientist, he could say almost nothing scientifically disciplined about what people were doing in all of these places.

Between 1947 and 1973, the Midwest team prepared, conducted and analysed a series of observations of children and an examination of the human habitat of their town and its environment. These initial studies led them to posit the existence of ‘behaviour settings’: standing patterns of behaviour within a physical and social milieu [56]. Settings are almost too obvious for us to notice, but they have more influence on behaviour than perhaps any other single thing. Examples include a coffee shop during opening hours, a church service, a mathematics lesson in a school classroom and a research seminar at a university. We spend almost all of our lives in such settings, and, as the research team would come to say, ‘People in school behave school, people in church behave church’.

The Midwest team did much more than just notice the presence of behaviour settings, however. Over the decades of the research station’s existence, they developed detailed models of how settings are structured, how they vary and constructed instruments (various checklists, for instance) to systematize their study.

Bequeathed to the behavioural sciences by this work is a large body of detailed, structured theory and methods appropriate to human behavioural ecology. It identifies core dimensions of behaviour settings, such as their physical, social and temporal characteristics. The theory characterizes different forms of social engagement within a setting, and how different events or forms of setting will impose constraints or demands on the people participating in it. The instruments developed provide a means to distinguish between a single setting with multiple action patterns, or multiple settings in the same physical location. Their research also models comparative work examining differences between locations for the provision and population of behaviour settings (e.g. big school, small school [59], and the comparisons between Midwest and Yoredale [60]). The theory also includes a host of hypothesized mechanisms by which the different dynamics of constraint and demand on inhabitants are brought into play.

All told, behaviour settings theory represents the most detailed and well-resourced account of behavioural ecology for human beings currently available. As such, it seems ideally placed to be integrated into a cognitive science that emphasizes situatedness and embedding in context. It is not surprising, then, that the past few years have seen something of a resurgence of behaviour settings in the cognitive science literature.

As Avram *et al*. [61] note, rates of citation for behaviour settings work have been on an upward trend through the twenty-first century to date. Several authors have endorsed behaviour settings as a promising means both of articulating and systematically examining the theorized integral relationship between person and environment that has come to prominence [62–66].

We, as researchers and of course editors of this theme issue, share this enthusiasm. There are a number of reasons to be optimistic about the value of behaviour settings work in the near term. Foremost among these reasons is the obvious power of behaviour settings to organize behaviour in real-world circumstances. This is something that has been exhaustively demonstrated by the Midwest team and those directly following them, but is also plain to see in common sense and intuitive terms. At a coarse grain of analysis, the single best predictor of any person’s behaviour at a given time is nothing that characterizes or concerns them as an individual but rather the behaviour setting in which they find themselves.

As demonstrated by several articles in the present collection, behaviour settings work also meshes with several aspects of critical reflection currently underway in the behavioural sciences. The theory and method have implications for evaluating the validity of psychological research in the real world (Baggs & Sanchez de Oliveira [7]; McGann [67]) and offer suggestions for grounding key concepts of embedded and situated cognition (Gastelum *et al*. [68]; Kalis *et al*. [69]).

Prospect in theory, however, has only limited value. As we have noted, behaviour settings research involves a number of specific and useful concepts, but it is not merely conceptual. The theory as developed by the Midwest team is exactingly operationalized (Avram *et al*. [61]; Lucas, [70]). It includes prescriptions for empirical work and guidelines for the organization of whole research agendas, including both surveying and mapping the human behavioural habitat, as well as prospects for broader social accounting [71]. The literature thus far has seen more enthusiasm for the theory than the method, and it is perhaps worth pausing to consider why.

## 3. Challenges to building on behaviour settings

We can identify three broad themes for the challenges faced by researchers engaging with empirical work directed at behaviour settings.

The first is the banal but non-negotiable reality of resource demands. While it is certainly possible to do valuable forms of empirical work on behaviour settings in the laboratory (e.g. [72]), the reality is that fully engaging with the phenomena means investigating them in the complex, poorly controlled and ethically fraught context of the ‘real world’. The demands of such work are always greater than those of behavioural science work undertaken in the laboratory, most significantly in terms of time, often the cost of equipment, and also additional administrative burden. The resource differential and concomitantly slower research outputs were some of the principal reasons that even alumni of the Midwest field station tended to transition into other forms of research when moving institutions [73]. Newly available technologies offer a great deal of hope in this area, simplifying the systematization of observation, recording and analysis. It remains the case, however, that the richer texture and greater validity of real-world research will always come at a premium compared to basic laboratory work.

The second, perhaps more interesting challenge also relates to changes in technology, though in this case, requiring the adaptation and extension of the original methods and theory of settings. The original empirical work was largely conducted in small, strongly traditional, almost idealized communities. Though there was some extension and comparison with more densely populated or urbanized settings, the kinds of situations observed by the Midwest team have changed dramatically in the intervening years. The past few decades have seen a transformation of life, work and social interactions brought about by digital technologies and accelerated in the past 4 years since the mammoth upheaval of the COVID-19 pandemic. Many, if not most, behaviour settings are transformed by the omnipresence of the internet. Setting dynamics may be diluted as participants are simultaneously in more than one setting—one in-person and another distracting them on their phones. It is also true that settings may no longer occur in a single physical location: they may be hybrid, mediated not just by local furnishings but also by intervening telecommunication technologies. Indeed, in some cases, they may also involve wholly simulated or fabricated locations, as virtual and augmented realities are woven into particular routines and practices.

Work has begun to address these changes in the nature of behaviour settings, including initial work on online activities as settings by Blanchard [74], and continued in the present collection, where Aunger *et al*. [75] present new tools and methodological techniques for mapping the relationships between different aspects of a behaviour setting’s hybrid milieu. The entire domain of cyberpsychology offers both intriguing possibilities and an interesting test case with respect to the limits of generalizability of theory around constraints and pressures offered by setting mechanisms. Given the increasing extent of online aspects of human behaviour ecology in much of the world (indeed, its near ubiquity in industrialized countries), it is vital that the limits and potential of settings methods and theory are much better understood than they are at present.

The third, and perhaps most critical challenge posed by behaviour settings work, concerns the relationship between settings and individual human beings. As a field, psychological science has something of a fraught relationship with the concept of the individual person generally [76–79]. As the Midwest work progressed, Barker in particular became convinced that behaviour settings research involved operating at a previously unrecognized scale of activity and required the emergence of a new field—that of ‘eco-behavioural science’ [80]. Despite this recognition, and the expansionist perspective on the agent–environment system that is characteristic of embodied, embedded and ecological cognitive science, it remains true that the individual perspective matters a great deal. Experience is famously something that occurs from a *first-person* point of view, and though agency is contextual, it is a phenomenon that nevertheless implies an agent: an autonomous individual making sense of, and adaptively coping with, their environment. While behaviour settings theory provides an account of that context, it is not clear how to integrate the perspective of the individual.

In the major publications summarizing the theory and methods of the Midwest team, Barker [56] and Schoggen [81], the role of people in settings is theorized in very passive terms. People are described as the *medium* for routines and practices of settings. The sense of medium in play is that of Heider [82], who characterizes the medium as fluid and responsive to the constraints and structure imposed by *things*, the stability of which is contrasted to the medium’s fluidity. The immense flexibility and adaptability of human behaviour, the skilful ways in which exceptions or unusual events are suppressed, resisted, managed or incorporated into the flow of a setting’s progress is part of what enables us to perceive the setting in the first place.

The tension between the conception of people as a passive medium and individuals as active agents is difficult to resolve. Indeed, the difficulty may be part of what underlies Barker’s recommendation for distinct scientific endeavours. Nevertheless, the need to reconcile these different perspectives is fundamental to providing a complete account of human behaviour and experience. We seem caught between two positions with strong empirical support. On the one hand, behaviour is clearly organized by the context in which we act; as already noted, behaviour settings are more predictive of people than any individual characteristic. On the other hand, the capability of individual action is the defining trait of psychology’s subject matter, and one of the most basic aspects of our experience as human beings.

We believe that there is reason for optimism, but also substantial work to be done.

Barker’s arguments for a distinct scientific endeavour were made at a time when the tools to integrate across scales were more limited than they are at present. The varied techniques drawn from the toolbox of complexity theory, dynamical systems theory and chaos theory provide a wealth of possibilities to look across scales, identifying tensions and resonances that produce fields of force for people involved [83–85]. In the present collection, Zieliński & Rączaszek-Leonardi [86] describe some tentative first steps in creating methods that bring the relationships between individual experience and behaviour setting to the fore, as well as how tensions in these arise, particularly in the case of differences of ability and expectations of ‘normality’.

Responses to this challenge will not only come in the examination of settings at the scale of the individual experience. Gastelum *et al*. [68], for example, outline some of the ways in which what are ostensibly within-person phenomena (in this case, inferences and reasoning) can be recognized as varying depending on the character of the setting in which the cognitive action is being undertaken. Indeed, such work seems entirely in keeping with a more general ‘social turn’ in reasoning research (most notably associated with the argumentative theory of Mercier & Sperber [87]).

## 4. The present issue

The collection of articles in the present issue has been prepared with the aim of presenting to the reader an effective and critical introduction to research on behaviour settings. This is done on the basis of clear evidence for the value of both theory and methods, but also in the knowledge that these remain relatively obscure in contemporary cognitive science.

The issue is roughly organized into three sections. The first orients the reader, providing a critical examination of the key concepts and methods of behaviour settings research. The extant body of work in the field is critically surveyed, with particular reference to work that is separate from and subsequent to that of the Midwest Psychological Field Station. Crucial to this review is the need to calibrate the technical vocabulary of behaviour settings theory. Benefits of the renewed enthusiasm for the approach will be maximized if we are careful to calibrate and deploy terms in a careful and precise manner. The second section includes a collection of articles that examine the relationships between behaviour settings work and contemporary concerns in cognitive science, while the final section addresses novel empirical work and professional practice informed and motivated by behaviour settings research.

Harry Heft [88], who has played a crucial role in re-invigorating behaviour settings theory in the modern era [89], opens the collection by offering an explanation for why Barker’s work on behaviour settings never took off originally and contrasts this with why now *is* the right time. Heft explains that although Barker’s work was initially acclaimed (receiving the American Psychological Association Award for Distinguished Scientific Contributions), the discipline more broadly was focused on studying either cognitive processes or the relationship between stimulus and response, often in cases where the stimulus was a highly impoverished representation of the environment. Over recent decades, there has been a slow but distinct metatheoretical shift in some areas of psychology with the emergence of *dynamical systems*, and key insights from *complexity theory*. In this light, Barker’s work should be appreciated anew. With this new framing, Heft discusses how the nested structure of behaviour settings has both top-down influences (higher-order collective patterns that influence individuals, such as historicity or ‘enabling constraints’ [65]) and influences from the bottom-up (how do individuals with agency create and shape behaviour settings?).

While Heft details the conceptual framework of behaviour settings theory, Avram, Jones, Lucas and Barrett [61] provide historical and empirical context of research in the domain since the work of the original Midwest team. These authors have conducted a systematic review of the literature and present a detailed overview of the current state of behaviour settings research. Not only do the authors describe the *‘*who, when, and where’ of behaviour settings literature (i.e. which disciplines are using it, the extent to which it is being used and where it is being used), but they also examine how it is being used. A key conclusion of this review is that the interdependence of the physical and the social, the organism and the environment emphasized by Barker is not as apparent as it might be, and the authors offer positive suggestions for how this may be remedied in the future.

It is precisely the measurement of interdependence with which Miranda Lucas [70] is most concerned. While many of the articles in this collection exemplify the value of the concepts of behaviour settings in orienting and organizing research on psychology in real-world settings, Lucas provides a much-needed worked example of the concrete practice of behaviour settings research. This article demonstrates the value not just of the theoretical orientation, but the tools and scales developed by the Midwest team, for the purposes of disciplining and refining our perceptions and observations of settings.

In the second part of the issue, this foundation of theories and methods is connected in various ways with contemporary concerns in the cognitive and behavioural sciences.

Perhaps most broadly, McGann [67] examines how some of the greatest innovations and most productive implications of the Midwest team’s work derives from their awareness of scale. McGann diagnoses a general problem of ‘scale-blindness’ in psychology: the manner in which psychologists employ theories that incorporate a range of scales (from rapid neural firing to prolonged shifts in cultural practices) without any explicit recognition that this is the case, and without offering any systematic means for dealing with such scalar variation. The result, unsurprisingly, is psychologists often confuse and conflate phenomena occurring at different scales, while remaining oblivious to having done so. At best, this means we have to deal with all kind of conceptual slippage and, at worst, we are faced with paradoxes and puzzles that simply cannot be solved. McGann shows how the Midwest team stand out in the history of psychology as being both explicit and disciplined with respect to engaging with the scale of their studies. He demonstrates why taking their views on board would benefit both scientific theory and practice, thus engaging with the enthusiasm for scientific reform currently extant in the psychological research community.

There are at least two other movements of reflection and reform within contemporary psychology, where behaviour settings theory is well positioned to make significant advances. First, behaviour settings theory has the potential to contribute to the methodological, theoretical and measurement reform efforts brought about in response to the so-called replication crisis in psychology [90]. Second, behaviour settings theory may help address the issue of WEIRD psychology, where most participants in psychology experiments are drawn from Western, Educated, Industrialized, Rich and Democratic societies [91,92]. While both are discussed, this second issue is a particular focus of Baggs and Sanches de Oliveira [7]. They describe four aspects of the so-called WEIRD crisis in psychology, including: (i) representativeness (non-generalizability owing to populations sampled), (ii) theory (assumptions of individualism and universalism), (iii) methods (reliance on artificial tasks, settings and measures), and (iv) infrastructure (publishing/funding opportunities, researcher diversity). Following this they discuss two programmes—behaviour settings theory and the distributed cognition programme [38]—and how each of the four aspects of the WEIRD crisis in psychology can be addressed by each programme.

The three other articles making up the second section address specific conceptual questions.

Sepúlveda-Pedro and Mojica [93] draw on the particular concepts of enactive cognitive science in order to examine the difficulty of conceiving the relationship between behaviour settings and the actions of individual participants. Articulating a common theme in enactive accounts they note how the successful enactment of behaviour settings depends on a balancing of tensions between the demands of the setting and the needs of the participants. In some circumstances, those tensions can result in a disruption or transformation of the setting. The enactive approach provides some guidelines as to what to look for, and in what aspects such tensions are likely to arise.

Kalis, Pascoe and Segundo-Ortin [69] examine what might be a specific example of such a tension, and one very commonly considered to be an inherently internal-only phenomenon. They show how behaviour settings theory can contribute to the development of a situated science of self-control (the ability to pursue goals in the face of conflicting motivations). Self-control is often construed in cognitivist terms, focusing on internal brain-based processes. In contrast, a situated account emphasizes the importance of the body and environment, as well as the brain, in explaining self-control. Kalis *et al*. go further, however, in offering the more precise claim that self-control cannot be explained in any kind of strictly internalist fashion: rather, it can only be ascribed to the person-in-context, and not to specific brain-based internal mechanisms at all (‘mental muscles’ or otherwise). In their view, self-control is a set of variable skills, whose exact nature will depend on the problem to be solved and the context in which it occurs. This, in turn, further implies that skilled self-control requires knowledge of which strategies will work best, and when to employ them. The situated part comes in once we understand that the structure of the environment plays a crucial role in skilled performance—people modulate their physical and social environments in ways that contribute directly to the successful pursuit of their goals. As such, self-control is a dynamic, interactive activity that spans brain, body and world.

The final example of a specific cognitive phenomenon reconsidered from a behaviour settings perspective is one that both addresses the process of reasoning in a situated manner and links the ecological psychology of Barker and colleagues with that of the more widely known Gibsons. The relationship between these perspectives is a nuanced one that needs to be approached carefully [89]. Nevertheless, there are clearly ways in which the two can enrich one another. Gastelum *et al.* [68] explore one such example, specifically how behaviour settings work can be deployed to better understand and conceptualize the process of ‘reasoning’. Reasoning is typically a paradigmatic example of a process that is ‘internal’ and computational, at odds with the ecological approach to understanding psychological processes. Gastelum *et al.* nevertheless demonstrate that the contexts of reasoning, and the resources made available by different behaviour settings for reasoning, play a significant role in what is taking place and how it should be understood.

The final group of articles each offer examples of novel empirical work informed by and extending behaviour settings theory and encounters with disciplines or settings beyond those addressed in the original research.

Di Rienzo *et al*. [94] tackle the issue of situational social norms, arguing that behaviour settings theory offers an alternative to the cognitivist view that normativity derives from internal rules and representations concerning the appropriate forms of conduct for a given situation. Rather than being ‘pre-formed’ as this view supposes, Di Rienzo *et al*. argue that normativity is, instead, *performed*: norms of behaviour are ‘out there’ in the social practices and material configurations of the situations that we encounter. This being so, there is no need to posit the existence of representations in our heads.

Di Rienzo *et al*. explore this idea using an observational study of a scientific laboratory. They illustrate both the reciprocity between the behaviour setting and the synomorphs (discrete physical furnishings or structures that support particular behaviours) that constitute it. The authors also explore how these are coordinated temporally and unfold over time. Specifically, they show how the standing patterns of behaviour of the laboratory experiment serve to organize the material environment into synomorphs that, in turn, organize the participants' activities by inviting them to maintain the setting.

Perhaps the most obvious change in the practicalities of real-world behaviour since the original Midwest research is the extent to which contexts for that behaviour are not just physical and social, but also virtual. The world we live in is arranged not just according to the logic and constraints of physics (including architecture and design) and social practice, but also according to the more configurable logics of software. Aunger, Deterding, Zhao and Baxter [75] offer tools to systematically incorporate virtual aspects of situations into behaviour settings research. They introduce two new tools—a ‘canvas’ and a settings ‘model’—to help visualize and evaluate the implications of settings that include both traditional and computationally produced aspects.

While others note how technologies have changed settings, Zieliński and Rączaszek-Leonardi [86] critically appraise how behaviour settings can and cannot provide guidance for understanding interactions between people within settings. The authors begin with considerations of assistive technologies, specifically for persons after laryngectomy, which make some kinds of action possible, but in a way that does not account for variation in style, tone or technique between different situations. Developing new and better tools requires a means of systematically considering and adjusting to context. The work of the Midwest team provides important first steps towards such goals, but the authors also identify clear limitations of the framework. In particular, they suggest that other complementary frameworks for observation are necessary to fully elucidate the nuances of human interaction in the environment.

Pedersen and Nielsen [95] also have an interest in design, but this time at the level of the whole setting. They delve into how understanding places as behaviour settings can alter the practices of architecture and design, as patterns of activity by inhabitants are taken up explicitly in the planning and design processes. They detail a case study of a new building for individuals experiencing homelessness. They emphasize the transition from a mere *place-for-being*—that is providing basic temporary necessities—to a more active *place-for-being-doing-acting*, where people are invited to engage meaningfully in activities that develop their personal agency. The authors explore how the design of behaviour settings can serve as enabling spaces to support users without creating patronizing environments, while breaking down perceived barriers to social services. They discuss design considerations for marginalized populations, focusing on institutional challenges and the goal of reducing health inequalities. The study draws on insights from a collaborative design process involving staff and users, highlighting the importance of inclusive design practices.

The final article provides a very closely related example of behaviour settings affecting practice. Awamleh [96] details the development of what he terms ‘Behaviour Setting Transformation Methodology’. This methodology applies insights from behaviour settings to the domain of architectural design. Importantly, the development of this methodology is presented as arising out of need, whereby limitations associated with conventional tools and practices were ‘ill-suited to adequately address the complexity and depth required to encompass the full spectrum of contextual and user considerations’. Awamleh describes the development of his alternative approach, which draws on behaviour setting theory to examine *how* spaces are actually used. The full benefit of this new approach is then explored by describing specific fieldwork where the method has been applied.

As Avram *et al*. [61] make clear, since its introduction, behaviour settings theory has never disappeared from the research literature. It has nevertheless remained obscure, playing little to no role in typical undergraduate training, not fitting well with the dominant theoretical paradigm, and problematic professional incentives (Heft [88]; Scott [73]). As the cognitive and behavioural sciences increasingly require an effective account of the behavioural ecosystem, this approach is now on the rise. The articles in the present collection form a connective tissue between the history and the future of behaviour settings research. This theme issue also constitutes the first step in developing new resources for the productive extension of behaviour settings work, and interested readers are encouraged to seek further information at http://www.behaviorsettings.org.

## Data Availability

This article has no additional data.

## Footnotes

^1^
‘You can drive Nature out with a pitchfork, but she keeps on coming back’. (transl. Simpon & Speake [17]).

## Dedication

We would like to dedicate this theme issue to all of the members of the Midwest Psychological Field Station, whose work continues to inspire.
